# A generalizable framework for genetic code expansion in bacterial systems beyond *E. coli*

**DOI:** 10.1128/mbio.01273-26

**Published:** 2026-09-28

**Authors:** Elise Van Fossen, Molly Stephenson, Andrew Frank, Soujanya Akella, Rowan Wooldridge, Andrew Wilson, Youngki You, Ernesto Nakayasu, Robert Egbert, Joshua Elmore

**Affiliations:** 1 Biological Sciences Division, Pacific Northwest National Laboratory 6865, Richland, Washington, USA; Institute of Microbiology, Beijing, China

**Keywords:** synthetic biology, chemical biology, lysine acetylation, cross-linking, noncanonical amino acids, protein engineering, genetic code expansion, tRNA, promoters

## Abstract

Genetic code expansion (GCE) enables the site-specific incorporation of noncanonical amino acids into proteins, providing a powerful platform to investigate and engineer biological functions with molecular precision. While GCE has facilitated transformative studies in *Escherichia coli*, its utility in bacteria other than *E. coli* has been hindered by the lack of universal, high-efficiency methods for its implementation and optimization. To address this limitation, we introduce a modular, extensible platform for the generalizable implementation and optimization of GCE in phylogenetically diverse bacteria, based on the host-agnostic serine recombinase-assisted genome engineering toolkit. We apply this approach to five bacterial species and demonstrate robust incorporation of structurally diverse noncanonical amino acids, including a key bacterial post-translational modification. This work provides a generalizable and scalable strategy to deploy expanded genetic systems in bacteria, advancing programmable microbial engineering across diverse applications.

**IMPORTANCE:**

Expanding the genetic code allows scientists to introduce new chemical functionalities into proteins, creating powerful opportunities to study and engineer biological systems. However, these technologies have remained largely confined to the laboratory bacterium *Escherichia coli* because adapting them to other bacterial species has been slow and organism-specific. We developed a general, chromosomally integrated framework that makes it easier to establish and optimize genetic code expansion across diverse bacteria. Using this approach, we identify practical design principles for improving performance, demonstrate transferability between distantly related species, and enable site-specific incorporation of modified amino acids into native bacterial proteins. This work expands access to advanced protein engineering technologies for studying bacterial post-translational regulation and developing engineered microbes for biotechnology, environmental applications, and synthetic biology.

## INTRODUCTION

Genetic code expansion (GCE) enables the site-specific incorporation of noncanonical amino acids (ncAAs) into proteins, increasing nature’s chemical repertoire and providing a powerful platform for investigating and engineering biological functions (1). The implementation of GCE in living organisms has enabled the construction of bioorthogonal protein-labeling systems (1–3), discovery of protein interactomes (4, 5), rewiring of enzymatic activities (6, 7), and incorporation of synthetic post-translational modifications into proteins (8, 9). In the model organism *Escherichia coli,* the utilization of genetic code expansion has uncovered basic scientific tenets (10–14), facilitated the creation of sophisticated ncAA-driven biocontainment systems (15), and generated strains with augmented functionality (6, 16).

Although transformative in model bacterial systems, such as *Escherichia coli*, because of the lack of efficient and universal methods for implementing and optimizing GCE across diverse bacterial species, the utility of GCE remains largely inaccessible to the broader microbial research community (17, 18). This limitation is especially significant given that non-*E*. *coli* bacterial species are increasingly studied in biomedical, environmental, and biotechnical contexts (19) for applications such as recombinant protein production (20–24), agricultural engineering (25–27), chemical production (28), and biomining (29–31). Many of the most promising microbial chassis for these applications are species that possess unique physiological traits or ecological advantages over *E. coli* and its close relatives (19).

In non-*E*. *coli* bacteria, GCE would enable a wide range of studies that range from highly challenging to impossible when using other techniques. Some examples include using ncAAs as post-translational modification mimics to interrogate lysine acetylation (a modification central to bacterial biology) (32), imaging in anaerobic or redox-constrained environments with compact fluorogenic ncAA tags (33), and minimally perturbative ncAA bioorthogonal click handles to probe secretion dynamics and biofilm formation in native cellular contexts (34). Beyond mechanistic studies, GCE platforms would empower the engineering of industrial and environmental strains through the incorporation of stability-enhancing ncAAs into key biocatalysts (16) and support the development of sophisticated biocontainment strategies by enforcing dependence on synthetic amino acids in genetically augmented microbes (15).

However, efforts to implement GCE in such organisms are hindered by the same obstacles encountered when synthetic biology tools developed in *E. coli* are ported to bacteria that are not closely related to *E. coli* (35, 36). These obstacles include limited genetic tractability, incompatibility with existing replicating plasmids, and specifically for GCE, the lack of host-agnostic, scalable strategies for optimizing the expression of translational machinery (17, 18). In this context, plasmid-based approaches, while widely used, present inherent limitations that hinder the reliable deployment of multi-component GCE systems in non-*E*. *coli* organisms. Replicating plasmids are frequently unstable, can impose considerable fitness burdens, and are constrained by a limited number of compatible plasmid backbones (37, 38). These issues are magnified when GCE requires distributing multiple components across different plasmids in microbes with very few available selection markers and few, if any, available replicating plasmids.

Here, we present a generalizable engineering framework that enables rapid implementation, optimization, and chromosomal deployment of genetic code expansion across bacterial hosts. Our platform leverages the serine recombinase-assisted genome engineering (SAGE) toolkit to achieve stable, site-specific chromosomal integration of multiple GCE components into diverse hosts at high efficiency (39). A key advantage of SAGE over plasmid-based systems and related technologies is that it does not require any DNA replication in the host, making it host-agnostic. Furthermore, SAGE directly fulfills two critical practical requirements: (i) sufficiently high transformation efficiency to construct and screen library-scale promoter variants that drive GCE components and (ii) strict modularity, allowing individual parts (promoters, tRNA/synthetase cassettes, reporters) to be swapped or re-optimized independently. SAGE supports iterative, marker-free, site-specific chromosomal integrations and has been explicitly demonstrated to enable pooled, genome-integrated promoter (39), ribosomal-binding site (40), and enzyme variant (41) libraries, making it well suited for high-throughput promoter optimization across diverse hosts. Its multi-site landing-pad architecture, comprising multiple orthogonal *att* sites and high-efficiency serine recombinases, provides the modular framework needed to install separate GCE components at defined loci and subsequently exchange or tune them without rebuilding entire strains. This platform combines the flexibility and efficiency of replicating plasmid-based systems with the genomic stability and reproducibility necessary for rigorous ncAA incorporation studies in hosts other than *E. coli* and related organisms.

To demonstrate the generality of this approach, we apply the workflow to five bacterial species spanning two phyla and incorporate eight structurally diverse noncanonical amino acids into proteins. As a stringent benchmark, we target Nε-acetyl-lysine, a ncAA with a subtle modification whose incorporation has not been demonstrated in bacteria beyond *E. coli* and its close relatives. While acetyl-lysine is a key bacterial post-translational modification, we use it here as a test case to assess system efficiency and flexibility. Using our optimization workflow, we increase the efficiency of acetyl-lysine incorporation from near-negligible levels to a level that is sufficient to encode ncAAs at multiple sites in endogenous proteins. In summation, this platform establishes a generalizable approach for rapidly onboarding and tuning GCE systems in *non-E. coli* microbes.

## RESULTS

### Multiplexed SAGE enables facile application of genetic code expansion in *Pseudomonas putida* KT2440

**Fig 1:**
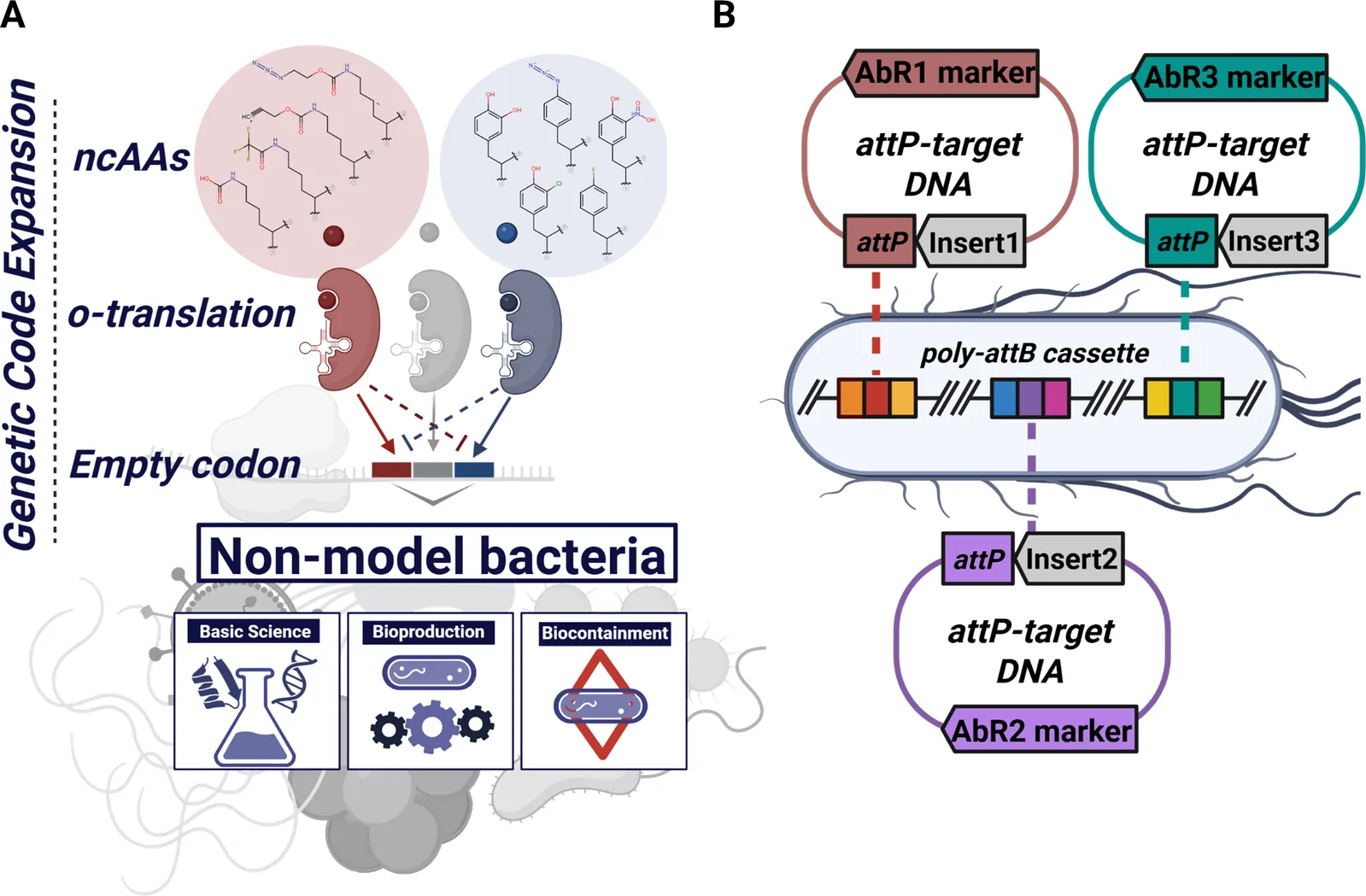
Overview of systems employed in this work. (**A**) Overview of genetic code expansion (GCE). Elements in red and blue represent engineered translational components that act orthogonally to endogenous translation (elements in gray) to produce site-specifically modified protein. (**B**) Overview of serine-recombinase-assisted genome engineering (SAGE), where serine-recombinases encoded on non-replicating plasmids facilitate the insertion of attP-containing target plasmids into cognate attB-sequences that have been installed into the host chromosome.

Implementation of plasmid-free genetic code expansion (GCE) requires integrating an orthogonal translation system into the host chromosome. At minimum, this system comprises three genetic elements: an orthogonal aminoacyl tRNA synthetase (o-aaRS), its cognate orthogonal tRNA (o-tRNA), and a target protein. The target protein carries a “blank” codon (typically a stop codon) at the desired site of ncAA incorporation (Fig. 1A). The orthogonal elements are engineered to recognize and “suppress” the stop codon with the ncAA to create full-length protein (42).

For our initial evaluation of the chromosomally integrated GCE system, we chose to incorporate the ncAA *para*-azido-L-phenylalanine (pAzF) using the *Methanococcus jannaschii* tyrosyl-tRNA synthetase/tRNA^Tyr^ (*Mj-TyrRS*/tRNA^Tyr^) system, specifically the *para*-cyano-L-phenylalanine aminoacyl-tRNA synthetase (*p*CNF-RS) (43). This system is a standard in bacterial GCE as it is highly efficient and polyspecific, recognizing at least 18 ncAAs, including *p*AzF, *p*CNF, and other *para*-substituted phenylalanine analogs. pAzF is particularly valuable because its biorthogonal azide handle supports both crosslinking and click chemistry-based labeling (44).

As chromosomally integrating each GCE component into a distinct *attB* site allows independent optimization, we separated the aaRS and its cognate tRNA onto separate constructs. This modular design allowed us to adjust tRNA availability by varying gene copy number and to tune aaRS expression via promoter strength, reducing tradeoffs that arise when both components share a single expression context. To implement this design, we used a variation of SAGE that allows integration of multiple distinct “target plasmids” into the chromosome without excising the antibiotic selection marker after each integration, as required in the original SAGE protocol (Fig. 1B).

Each GCE component was linked to one of three antibiotic selection markers and serine recombinase pairs, enabling chromosomal integration of each element in any order. For this method, we built target plasmids containing each of the three required GCE components with unique antibiotic resistance markers and distinct *attP* sites. These *attP* sites direct integration into three different *attB* sites in the bacterial chromosome via three corresponding serine recombinases. Specifically, the *Mj-TyrRS* was targeted to the TG1 *attB* site, the *Mj-*tRNA^Tyr^ to the Bxb1 *attB* site, and the protein of interest to the R4 *attB* site (Fig. S1B).

We first tested the SAGE-based GCE system in *Pseudomonas putida* KT2440 (45, 46). *P. putida* is increasingly used for industrial and environmental applications (47) due to its robust redox metabolism, stress tolerance, rapid growth, metabolic versatility, and nonpathogenic nature. Recently, replicating plasmid-based GCE was reported in *P. putida* for the first time (48), motivating us to use it as a testbed for SAGE-based GCE. We employed a SAGE-compatible strain of *P. putida* KT2440 (AG5577) as the basis of all subsequent GCE work in *P. putida*. This strain contains nine distinct heterologous *attB* sites, each recognized by a different serine recombinase from the SAGE toolkit (39).

**Fig 2:**
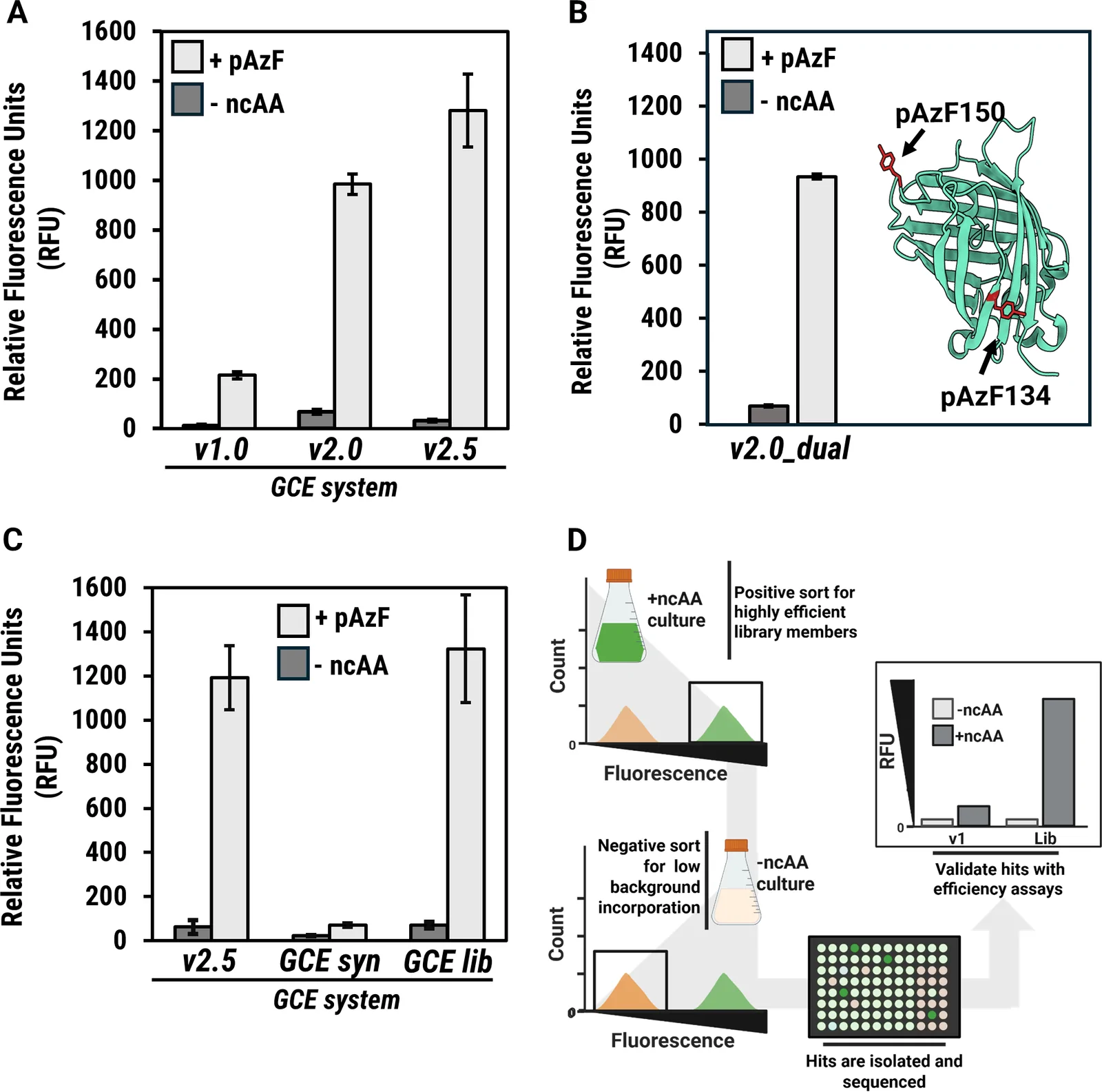
GCE optimization strategies in *Pseudomonas putida* KT2440. (**A**) GCE efficiency assays comparing each version of the GCE system. The GCE system used is indicated on the *x*-axis. The presence (light gray) or absence (dark gray) of pAzF in the medium is indicated. (**B**) Overview of dual-incorporation system. GCE incorporation efficiency for the dual-incorporation system is shown on the right. The structure of sfGFP is also shown, with each incorporation site highlighted in red. (**C**) GCE efficiency assays comparing the synthetic GCE translational system and a representative member of the sorted GCE library. Error bars in panels **A–C** represent the standard deviation in four biological replicates. (**D**) Schematic of the FACS-based library selection for promoter optimization. After transformation into KT2440, the promoter library is cultured in the presence of ncAA to elicit a fluorescent signal. Fluorescent cells are sorted by FACS and then cultured in the absence of ncAA. Individual cells with low fluorescence in the absence of ncAA are then sorted for sequencing and validation.

Multiple studies implementing GCE in bacterial hosts other than *E. coli* have reported that regulatory elements (promoters, ribosome binding sites, etc.) often require host-specific optimization (17, 18, 34, 49). To maintain physiologically relevant expression levels in *P. putida*, we followed a previously reported strategy (48, 49). We identified the most abundant codon in the *P. putida* genome (CUG, encoding leucine) and the corresponding aaRS and tRNA. The native promoters and terminators flanking tRNA^Leu^ and leucyl-tRNA synthetase (LeuRS) were used to drive expression of the *Mj-*tRNA^Tyr^ and *Mj-TyrRS*, respectively, to create version 1.0 (v1.0) of the system (Fig. S1B). After integrating all GCE components into the *P. putida* chromosome, we evaluated ncAA incorporation efficiency using a stop codon readthrough assay with superfolder GFP (sfGFP) as a reporter (50). Going forward, we refer to these as GCE efficiency assays. In this reporter construct, sfGFP carries an amber stop codon at position N150 (sfGFP_TAG150_). In the absence of ncAA incorporation, expression produces a truncated, non-fluorescent protein. Successful ncAA incorporation at the amber codon yields full-length, fluorescent sfGFP, allowing cellular fluorescence to serve as a direct proxy for ncAA-containing sfGFP production. Consistent with prior work in *P. putida*, the pCNF-RS system enabled efficient incorporation of pAzF, as indicated by moderate full-length sfGFP expression only in the presence of pAzF, confirmed by top-down protein mass spectrometry (MS) (Fig. 2A; Fig. S2).

Although this initial system successfully incorporated pAzF, the efficiency remained suboptimal. As high o-tRNA concentrations are often required for robust incorporation in other GCE systems (51), we hypothesized that increasing o-tRNA copy number would enhance ncAA suppression. To test this, we designed a tRNA cassette modeled on an endogenous *P. putida* tRNA operon that encodes two identical tRNA^Leu^ in a single pre-tRNA transcript and used this for version 2.0 of the system (Fig. S1A and C). Our cassette uses the native tRNA^Leu^ promoter, the inter-tRNA spacer, and the downstream terminators but replaces each tRNA^Leu^ with *Mj-tRNA*^Tyr^. We also identified a native tRNA operon comprising three tRNAs located upstream of an elongation factor protein (EF-Tu) gene, which encodes the protein that delivers aminoacyl-tRNAs to the ribosome. As native EF-Tus can be less efficient with ncAAs and orthogonal translation components, we drew inspiration from this operon and placed a pAzF-optimized EF-Tu (52) downstream of the dual *Mj-tRNA*^Tyr^ operon described above, generating version 2.5 (v2.5, Fig. S1C). Finally, we streamlined v1.0 by combining the sfGFP_TAG150_ reporter and *Mj-TyrRS* on a single construct (Fig. S1C).

We evaluated the incorporation efficiencies of the upgraded GCE systems (v2.0 and v2.5) and found that adding a second copy of *Mj-tRNA*^Tyr^ (v2.0) increased incorporation ~5-fold relative to the original single-tRNA system. Adding the engineered EF-Tu in v2.5 provided a further ~1.5-fold improvement (Fig. 2A). Overall, pAzF incorporation improved from ~6% production of pAzF-sfGFP relative to WT sfGFP to 57% with v2.5 (Fig. S3).

To further probe system performance, we designed an sfGFP variant bearing two amber codons (sfGFP_2XpAzF_; D134TAG and N150TAG) for dual pAzF incorporation (Fig. 2B). Using v2.0, we observed robust fluorescence, indicating reasonable dual-incorporation efficiency and demonstrating that the system can support multiple ncAA incorporation events (Fig. 2B). Top-down protein MS confirmed dual pAzF incorporation, with slight degradation during purification accounted for in the mass calculation (Fig. S4).

### SAGE enables high-throughput library-based GCE optimization directly in the host organism

Multi-component systems, such as GCE, often require coordinated tuning of each element for optimal performance (53). A common strategy is to adjust promoters and 5′ UTRs (including ribosomal binding sites) to modulate transcription and translation. Although the native LeuRS promoter supported strong performance in v2.0, its poorly defined RNA polymerase binding motif limited further rational engineering for altered expression. The synthetic *tac* promoter is widely used in bacteria, offers strong base expression, and is easily tunable through modification of its well-characterized core promoter elements (54, 55). We therefore evaluated the *tac* promoter as a replacement for the LeuRS promoter to drive *Mj-TyrRS* expression in *P. putida* (GCE syn system, Fig. S1D). Unexpectedly, sfGFP_TAG150_ expression decreased substantially when *Mj-TyrRS* transcription was driven by tac rather than the native LeuRS promoter (Fig. 2C), consistent with prior observations that GCE machinery originally optimized in *E. coli* requires careful retuning in other hosts (18).

Instead of individually testing large numbers of promoter/ribosomal binding site (RBS) variants, we leveraged SAGE’s high transformation efficiency and the sfGFP reporter to screen a large *Mj-TyrRS* expression library in a pooled format. We previously showed that SAGE supports high-throughput, sequencing-based assessment of promoter libraries in pooled assays (39). Here, we applied a similar strategy to identify regulatory elements that provide sufficient *Mj-TyrRS* for robust ncAA incorporation while maintaining orthogonality.

We constructed a pooled *Mj-TyrRS* expression library using 449 synthetic and natural promoters (Table S1). To span a wide range of translational strengths, we randomly cloned one of 106 RBS variants downstream of each promoter. These RBSs were designed using an RBS calculator (56) to cover a ~441-fold range in predicted translational initiation rates (Table S2). Each promoter–RBS module also included a RiboJ insulator (57) to stabilize expression. The resulting library (~209,000 unique promoter–RBS combinations) was integrated into *P. putida* using SAGE, yielding a pooled collection of ~10^7^ transformants. Each cell contained a distinct promoter–RBS pair driving *Mj-TyrRS* expression, along with the sfGFP_TAG150_ reporter and the dual o-tRNA cassette from v2.0 (Fig. S1E).

Because GCE efficiency is reported by sfGFP fluorescence, we used fluorescence-activated cell sorting (FACS) to select variants that were both efficient and orthogonal (Fig. 2D). The library was first grown in the presence of pAzF to allow for ncAA-dependent sfGFP production. Cells with high fluorescence, indicating efficient ncAA incorporation, were enriched by a positive FACS sort (top 1.5% most fluorescent events). This enriched pool was then grown in the absence of pAzF and subjected to a second sort, this time selecting cells with fluorescence at or near background, thereby enriching for variants with low misincorporation of canonical amino acids. Individual low-fluorescence clones were plate-sorted, grown in microtiter plates, sequenced, and further validated in scaled GCE efficiency assays (Fig. S5; Tables S1 through S3).

The top-performing strain displayed ncAA incorporation efficiency and orthogonality comparable to v2.5 (Fig. 2C), despite lacking an engineered EF-Tu. Surprisingly, this strain used a Lactobacillus_39770 promoter that exhibits relatively low transcriptional activity in *Pseudomonas* species (Table S3) (39). In our prior work, this promoter showed between ~24- and 92-fold lower activity than the *tac* promoter (39), suggesting that poor performance of *tac* in this context likely represents excessive *Mj-TyrRS* expression.

In contrast, the selected strain paired this lower-activity promoter with an RBS variant having a high predicted RBS translation initiation rate (67,067.6), comparable to that in *tac-*driven system (Table S2). Thus, a single round of positive and negative FACS selection yielded a promoter–RBS combination that matches or exceeds the performance of native regulatory elements, demonstrating that non-native promoters can be rationally identified to drive efficient, orthogonal GCE in bacteria other than *E. coli*.

### SAGE facilitates the genetic code expansion of phylogenetically distant microbes

**Fig 3:**
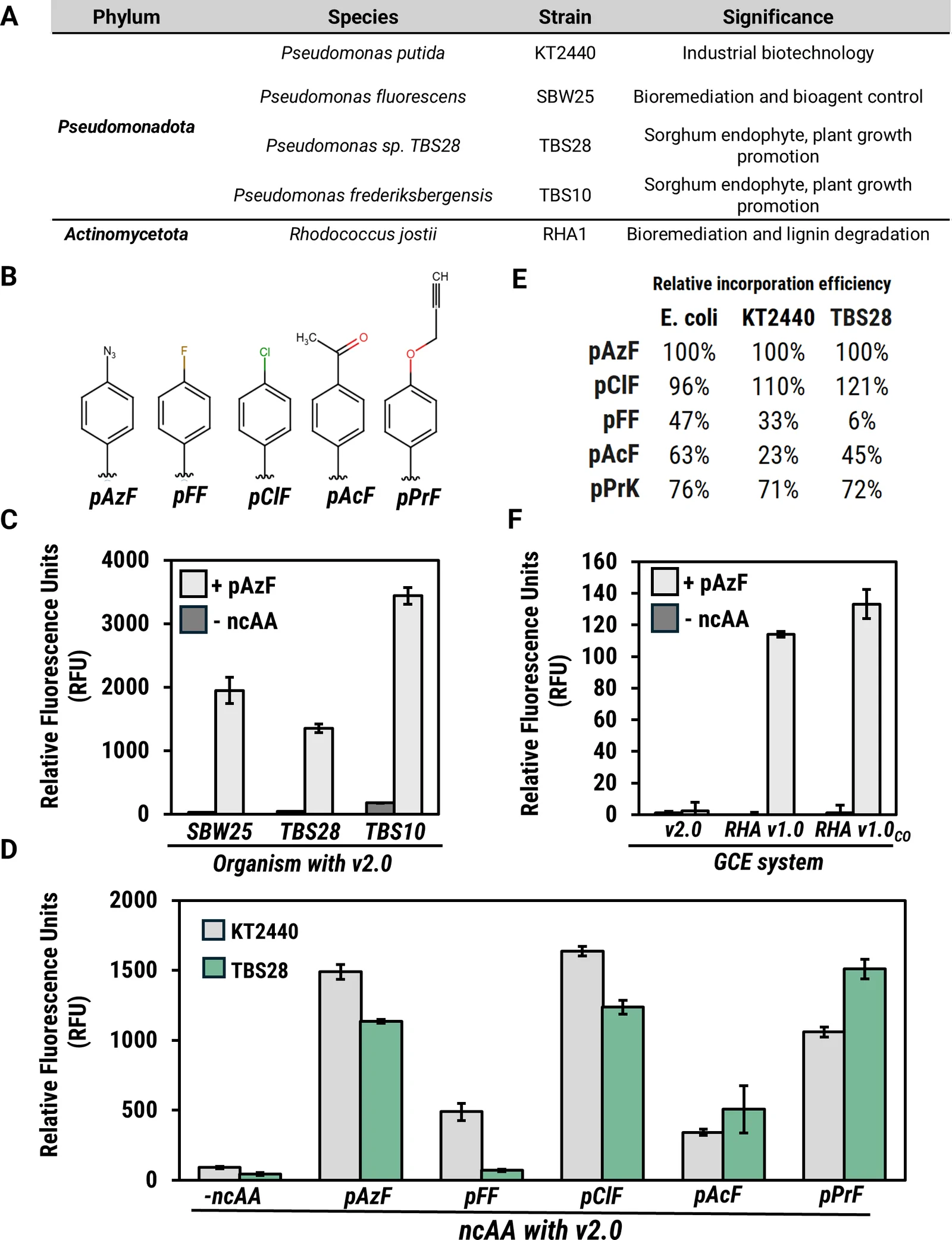
GCE-SAGE technology in non-*E. coli* bacteria. (**A**) Overview of bacterial species used in this study. (**B**) Structures of ncAAs screened in KT2440 and TBS28 strains. (**C**) GCE efficiency assays for GCE v2.0 in three pseudomonads. The pseudomonad used is indicated below each column set on the *x*-axis. The ncAA condition for each system is disclosed in a legend in the upper left of the figure. (**D**) GCE efficiency assay for KT2440 and TBS28 with GCE v2.0. The ncAA used is indicated below each column set on the *x*-axis. The strain is disclosed in a legend in the upper left of the figure. (**E**) The incorporation efficiency of each ncAA relative to pAzF is shown for *E. coli*, KT2440, and TBS28. (**F**) GCE efficiency assays for GCE v2.0 vs GCE RHA in RHA1. The GCE system used is indicated below each column set on the *x*-axis. The ncAA condition for each system is disclosed in a legend in the upper left of the figure.

After successfully implementing GCE in *P. putida* KT2440, we next tested how easily our optimized systems could be transferred to related *Pseudomonas* species. We selected the plant growth-promoting rhizobacterium *P. fluorescens* strain SBW25 (58) and two *Sorghum bicolor* root endophytes: *P. frederiksbergensis* (TBS10) (39) and *Pseudomonas* sp. TBS28 (59) (Fig. 3A). Both TBS10 and TBS28 had been previously engineered to be SAGE-compatible via chromosomal integration of a poly-*attB* landing site (39, 59).

We directly ported the v2.0 GCE machinery from *P. putida* into the chromosomes of these three *Pseudomonas* strains and observed pAzF incorporation efficiencies comparable to those in *P. putida*, demonstrating that SAGE-based GCE systems can be readily shared among closely related bacteria (Fig. 3B andC ). Although GCE efficiency was similar, absolute fluorescence varied by ~2-fold across strains, potentially reflecting differences in gene expression, ncAA uptake, or other physiological traits.

Because *p*CNF-RS is polyspecific, it can charge the o-tRNA with multiple *para*-substituted phenylalanine analogs. However, each host encodes a distinct set of transporters and regulators that influence ncAA uptake and intracellular availability, and the contribution of these factors to GCE performance is not well understood. To probe this, we exploited the permissivity of pCNF-RS (43) and screened four additional *para*-substituted phenylalanine derivatives in both *P. putida *and *Pseudomonas* sp. TBS28 (Fig. 3B), then compared their relative incorporation efficiencies to published values in *E. coli* (43) (Fig. 3D and E).

Overall, the relative incorporation trends were similar across the three organisms, but notable differences emerged. Both pseudomonads showed reduced incorporation of 4-fluoro-L-phenylalanine compared with *E. coli*, and incorporation of this ncAA was nearly absent in *Pseudomonas* sp. TBS28 (Fig. 3E). Given that other ncAAs were incorporated with similar efficiencies, these differences are unlikely to arise from altered pCNF-RS activity and more likely reflect differences in amino acid transport, emphasizing the importance of considering ncAA import mechanisms when extending GCE to new bacterial hosts.

A key advantage of SAGE-based tools is their portability across phylogenetically distant SAGE-compatible bacteria. To demonstrate this, we tested our *P. putida* GCE 2.0 system in the actinomycete *Rhodococcus jostii* RHA1 (60), a representative of a genus with broad metabolic capabilities, including catabolism of cholesterol (61), petroleum-derived hydrocarbon (62), and lignin-derived aromatic compounds (63). When we transferred v2.0 into RHA1, ncAA-dependent sfGFP fluorescence remained at background levels (Fig. 3F), indicating that the *Pseudomona*s-optimized system was not directly compatible with this host.

To improve performance in RHA1, we applied the same host-tailoring strategy used in *P. putida*. We identified an alanine codon as the most abundant codon in the RHA1 genome and used the native promoters associated with the alanine tRNA and its aminoacyl-tRNA synthetase to drive expression of the GCE components (Fig. S1F). Given the high GC content and strong codon bias in *Rhodococcus*, we also generated a codon-optimized version of the protein-coding GCE components to test whether codon usage further influenced efficiency (Fig. S1F). The RHA1-tailored system showed a very large improvement over the directly ported v2.0, with only modest differences between codon-optimized (RHA v1.0_CO_) and non-optimized (RHA v1.0) variants (Fig. 3E). Nonetheless, the overall fluorescence remained comparable to v1.0 in *P. putida*, indicating room for further optimization.

### Site-specific incorporation of acetyl-L-lysine and other lysine analogs

Encouraged by the performance of the *Mj-TyrRS*-pCNF system, we next used our platform to expand lysine post-translational modification (PTM) tools for use in bacteria other than *E. coli*. As the *Mj-TyrRS* system primarily incorporates aromatic ncAAs, we turned to the pyrrolysine (Pyl) system. The Pyl system consists of a pyrrolysyl-tRNA synthetase (PylRS) and its cognate tRNA (tRNA^Pyl^) and supports site-specific incorporation of lysine analogs due to the structural similarity between pyrrolysine and lysine (64, 65).

**Fig 4:**
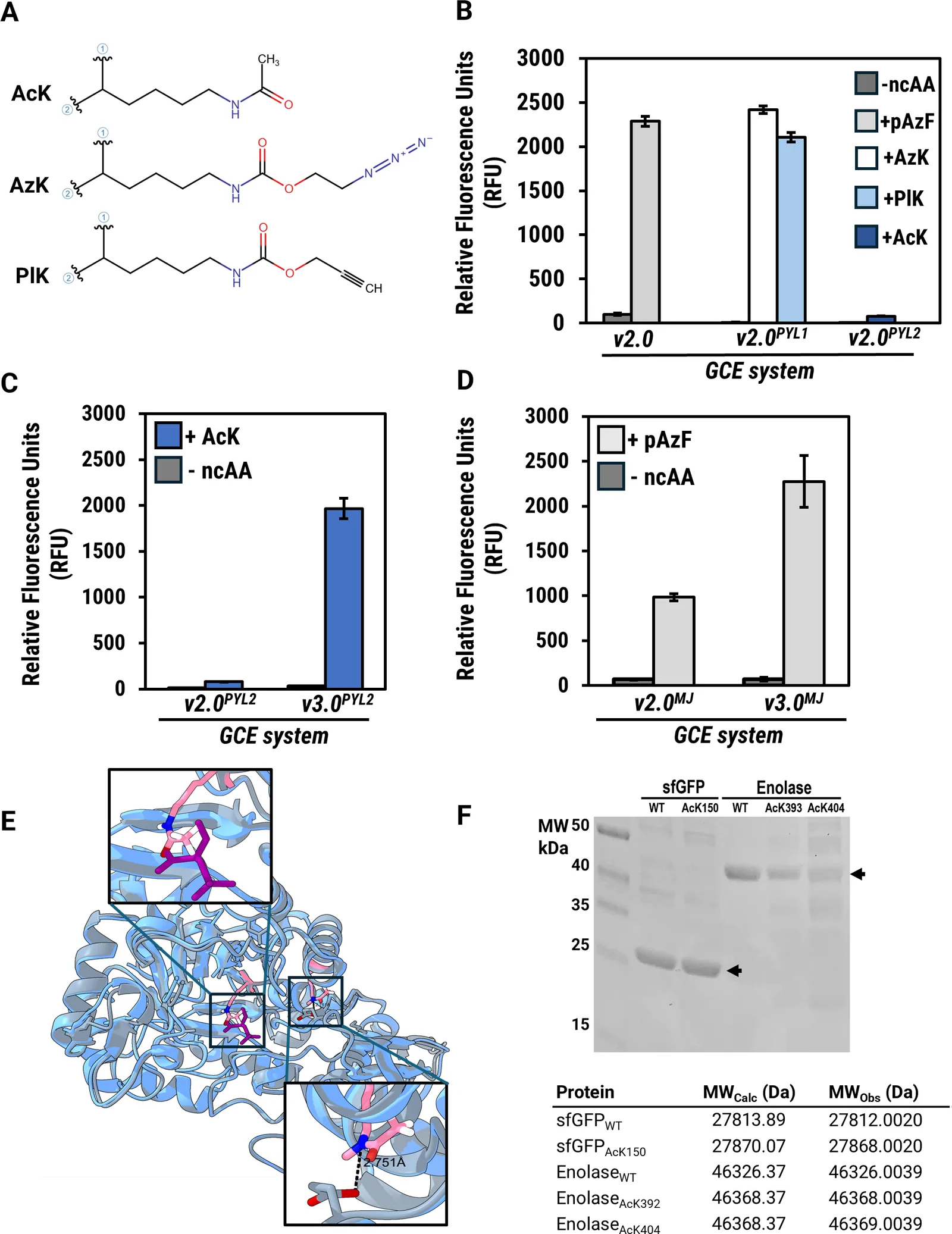
SAGE-based optimization permits facile acetyl-lysine incorporation. (**A**) Structures of ncAAs incorporated with Pyl components. (**B**) GCE efficiency assays with Pyl systems in KT2440. The GCE system used is indicated below each column set on the *x*-axis. The ncAA condition for each system is disclosed in a legend on the right of the figure. (**C**) GCE efficiency assays comparing the GCE v2.0 and GCE v3.0 Pyl systems. (**D**) GCE efficiency assays comparing the GCE v2.0 and GCE v3.0 Mj systems. For both panels **C and D**, the GCE system used is indicated below each column set on the *x*-axis. The ncAA condition for each system is disclosed in a legend in the top left of the figure. (**E**) Alpha fold-generated structure of KT2440 enolase with acetylations modeled onto relevant lysine residues (blue ribbon structure) overlaid on PDB structure of enolase from *E. coli* (gray ribbon structure) with bound substrate 2PGA (purple molecule). (**F**) Top panel: SDS-PAGE of acetylated proteins, purified from KT2440. Arrows indicate the MW for each set of purified proteins. Bottom panel: observed intact masses of purified acetylated proteins with their respective calculated masses.

We selected three lysine analogs for incorporation into *P. putida*: acetyl-lysine (AcK), azido-lysine (AzK), and propargyloxycarbonyl-lysine (PlK) (Fig. 4A) (65). AcK allows direct interrogation of how lysine acetylation affects protein function *in vivo* and *in vitro* (65), whereas AzK and PlK provide distinct bioorthogonal handles (66) for probing AcK-dependent protein interactions, fluorescent labeling, and, in some cases, site-specifically modeling of ubiquitin (42).

We integrated the Pyl system into *P. putida* using the same genetic framework as v2.0. Two engineered PylRS variants were used: *Mb-*PylRS-AF (PylRS1), containing active-site mutations that accommodate bulky lysine analogs (67) suitable for AzK and PlK; and chPylRS-IPYE (PylRS2), a chimeric synthetase reported to efficiently incorporate AcK in *E. coli* (68). Both synthetases were paired with an evolved tRNA^Pyl^ that enhances AcK incorporation (69) (Fig. S1G).

With PylRS1 in the v2.0 framework, we observed a high incorporation efficiency of AzK and PlK without further modifications (Fig. 4B). In contrast, AcK incorporation via PylRS2 was very low in *P. putida* (Fig. 4B). Combined with the scarcity of reports of AcK incorporation outside *E. coli*, this suggests that AcK is intrinsically more challenging to incorporate than many other ncAAs.

As we had found that increasing *Mj-tRNA*^Tyr^ copy number improved pAzF incorporation, we hypothesized that the tRNA^Pyl^ copy number might likewise limit AcK incorporation. To test this, we added an additional dual tRNA^Pyl^ operon at a third *attB* site in *P. putida* (v3.0; Fig. S1G), increasing the total tRNA^Pyl^ copy number from two to four. We applied the same modification to the *Mj-TyrRS*-pCNF system then and measured ncAA incorporation efficiencies for both.

Doubling the tRNA copy number improved incorporation in both systems, but to markedly different extents. For the already robust *Mj-TyrRS* system, sfGFP production increased ~2-fold upon addition of the second tRNA cassette (Fig. 4C). By contrast, AcK incorporation via chPylRS(IPYE) increased ~25-fold (Fig. 4D), bringing AcK incorporation efficiency up to the level of our best existing systems. These results support that tRNA expression can be a major limiting factor and a powerful optimization lever for GCE in each host.

### Production of site-specifically acetylated enolase in *Pseudomonas putida* with genetic code expansion

We were encouraged by the substantial improvement in GCE efficiency achieved by increasing tRNA copy number but sought to further validate the system by producing an essential protein bearing acetyl-lysine at biologically relevant residues. Nakayasu et al. previously performed a proteomic analysis that identified acetylation of highly conserved lysines in central metabolic enzymes from diverse bacteria, including *P. putida* ( 70). One such protein was enolase, an essential enzyme in glycolysis and gluconeogenesis, that catalyzes the reversible conversion of 2-phosphoglycerate to phosphoenolpyruvate (70). Beyond its metabolic role, enolase has moonlighting functions linked to bacterial stress responses and pathogenicity (71). We therefore selected enolase as a model to test AcK incorporation at multiple conserved lysine residues.

The prior study highlighted two conserved lysines of interest: K392, buried within the active site and involved in phosphoenolpyruvate catalysis, and K404, positioned near the protein periphery (Fig. 4E). Under the conditions tested by Nakayasu et al., K404 was acetylated in *P. putida*, whereas K392 (whose acetylation can abolish enolase activity) was observed to be acetylated in other bacteria but not in *P. putida* (70).

*P. putida* encodes multiple lysine deacetylases, including those CobB and metal-dependent deacetylases (NCBI: txid160488), which often act on a broad range of substrates, but do not necessarily deacetylate all acetyl-lysine sites. We hypothesized that the absence of detectable acetylation at K392 might result from efficient site-specific deacetylation, rather than from a lack of acetylation. By producing enolase with site-specific AcK *in vivo* in *P. putida*, we could assess whether acetylation at each position is maintained over the course of a typical cultivation. Stable acetylation would argue against active removal at that site under these conditions and instead suggest that K392 simply was not acetylated in the original proteomic experiments.

To generate site-specifically acetylated enolase, we replaced the gene for sfGFP_TAG150_ in the v3.0 framework with three enolase constructs in *P. putida*: wild-type enolase (enolase_WT_), enolase_TAG392_, and enolase_TAG404_. Cultures for strains harboring each construct were grown to stationary phase, after which enolase was extracted and affinity-purified for expression and acetylation analysis. All the three variants were successfully expressed as confirmed by SDS-PAGE (Fig. 4F, top panel), and intact molecular masses were determined by mass spectrometry. For enolase_TAG392_ and enolase_TAG404_, the measured masses were consistent with AcK incorporation at corresponding sites (Fig. 4F, bottom panel, Fig. S6 and S7). Although other contaminating proteins were detected, a mass associated with enolase_WT_ was not observed in the acetylated enolase samples (Fig. S6). While not exhaustive, these data argue against intrinsic lysine deacetylase activity during cultivation as the cause of the previously observed lack of K392 acetylation.

## DISCUSSION

Genetic code expansion (GCE) provides powerful control over protein structure and function (1), but its use beyond *Escherichia coli* has been constrained by a lack of generalizable, high-efficiency implementation strategies. Host-specific differences in genetic architecture, codon usage, and physiology, especially in industrially and environmentally relevant bacteria that are not closely related to *E. coli*, make it challenging to port and optimize GCE systems across diverse microbial strains.

Here, we address this barrier by developing a SAGE-based workflow for chromosomal implementation and optimization of GCE that is readily transferable across diverse bacterial hosts (39, 41). Whereas previous studies have focused on establishing or characterizing GCE within individual organisms (18, 48, 72), the SAGE platform provides a modular framework for rapidly onboarding and tuning GCE in new bacterial species using a common set of genetic tools.

Multiplex SAGE enables plasmid-free, site-specific, and stable integration of multiple GCE components into distinct landing pads in the chromosome. Once built, genetic cassettes can be reused across distantly related microbes, including multiple classes of proteobacteria (39, 41, 59) and actinobacteria (40, 41, 73), greatly reducing the effort required to deploy powerful genetic tools in new hosts. A practical consideration when implementing SAGE in a new host is the initial installation of a chromosomal attB landing pad, which requires a host-compatible genome engineering method such as allelic exchange, recombineering, transposon-mediated integration, or another targeted integration strategy. This one-time genetic manipulation is a prerequisite for SAGE cycling; however, once established, subsequent genomic integrations are independent of host recombination machinery and do not require replicating plasmids (39). As a result, iterative strain engineering becomes operationally similar to routine plasmid transformation while providing the advantages of stable chromosomal integration.

We chose SAGE in the context of other available chromosomal integration platforms. Chassis-independent recombinase-assisted genome engineering (CRAGE) enables single-step insertion of very large DNA payloads into many strains beyond *E. coli* and is excellent for installing entire biosynthetic gene clusters (74). However, its transformation efficiencies are often too low for library-scale workflows, and the reliance on one or two landing pads per strain complicates independent tuning of multiple small, interacting expression cassettes. CRISPR-associated transposase (CAST)-based tools offer programmable, RNA-guided insertion without double-strand breaks and are in principle well suited to library generation (75). In practice, CAST systems often require host-specific optimization for reliable performance, and high-throughput, pooled library workflows remain less mature than those established for SAGE or CRAGE. For our goal, modular, multi-locus integration, and pooled library optimization across diverse bacteria, SAGE currently offers the most practical combination of robustness, efficiency, and flexibility.

Here, we demonstrate this to enable GCE in two distinct bacterial phyla. Together with our related work applying the same general strategy to multiplex CRISPR interference in phylogenetically distant bacterial genera (40), this highlights the broad extensibility of the approach for both adapting existing tools to new species and introducing entirely new capabilities into non-model hosts. Critically, the same framework also supports rapid and parallel optimization of individual GCE components using either targeted constructs or large genetic libraries, streamlining host-specific tuning of ncAA incorporation systems.

Using *Pseudomonas putida* as an initial testbed, we found that GCE performance is sensitive to expression of its components and that SAGE greatly simplifies expression optimization. Consistent with previous studies, increasing orthogonal tRNA copy number substantially improved ncAA incorporation, particularly in initially modest systems, and this effect was reproducible across multiple ncAAs and tRNA/synthetase pairs. Because tRNA genes are easy to duplicate and distribute across *attB* sites, tRNA abundance emerges as a simple, generalizable lever which can be readily employed within the SAGE framework for tuning GCE in hosts that are evolutionarily distant from *E. coli*. In parallel, we used SAGE’s high transformation efficiency to perform pooled, fluorescence-based screening of a promoter/RBS library driving *Mj-TyrRS* expression, identifying regulatory elements that balance efficient incorporation with high orthogonality and revealing that overexpression of orthogonal components can be detrimental. Finally, portability tests, such as moving an optimized cassette into additional *Pseudomonas* strains and then into the actinomycete *Rhodococcus jostii*, showed that while closely related hosts required minimal retuning, more distant hosts needed re-engineered regulatory elements to regain robust functionality.

Although the orthogonal translation systems used in this study exhibited minimal reporter expression in the absence of the supplied noncanonical amino acid, indicating high amino acid specificity, comprehensive characterization of orthogonality in a new host should also include evaluation of off-target suppression of endogenous stop codons. These results demonstrate that GCE component expression must be empirically tuned for each host, and that SAGE provides a practical, reusable framework for carrying out this optimization across diverse bacteria. Our comparative screening of para-substituted phenylalanine analogs across related species revealed differences in ncAA incorporation that are likely driven by host-specific transport and uptake rather than synthetase activity. Such transport effects are an often overlooked barrier to GCE deployment in new hosts. Because SAGE can also be used to integrate heterologous transporters, sensors, or regulatory modules, the same framework can be extended to systematically evaluate and enhance ncAA uptake in recalcitrant organisms.

We also implement a pyrrolysine-based system to incorporate lysine analogs, including acetyl-lysine, into *P. putida*. Despite strong prior performance in *E. coli*, initial acetyl-lysine incorporation was nearly undetectable. These results, and the lack of studies demonstrating AcK incorporation outside of bacteria not closely related to *E. coli,* indicate that AcK incorporation can be challenging in non-*E*. *coli* bacterial hosts. Using an optimized platform, we site-specifically acetylated the essential enzyme enolase at conserved lysines K392 and K404 and confirmed AcK incorporation by mass spectrometry. The absence of unmodified enolase in these samples argues against pervasive deacetylation, illustrating how GCE can be used to interrogate native post-translational regulation directly in the host.

More broadly, this platform enables systematic interrogation of post-translational modifications in living bacteria, providing a route to mechanistically link specific amino acid modifications, such as acetylation, to cellular physiology (76). Beyond fundamental biology, tunable GCE systems could be used to engineer biocontainment safeguards by coupling essential functions to ncAA availability. The ability to stably implement and optimize GCE in a wide range of bacterial hosts also opens opportunities to dissect complex, multi-protein processes, such as secretion pathways, where site-specific chemical modifications can be used to probe dynamic interactions.

Together, these results establish a generalizable framework for stable implementation and flexible optimization of GCE in phylogenetically diverse bacteria. The SAGE-based platform is host-agnostic, modular, and compatible with scalable library workflows, substantially lowering the barrier to deploying ncAA technologies in bacteria that are not closely related to *E. coli*. In doing so, it broadens the scope of programmable protein engineering and accelerates efforts to design microbial systems with novel chemical functionalities for fundamental research and biotechnology.

## MATERIALS AND METHODS

### Reagents and media

All commercial chemicals are of reagent grade or higher. All solutions were prepared in deionized water that was further treated by Barnstead Nanopure ultrapure water purification system (Thermo Fisher Scientific). *Pseudomonas putida* AG5577, a derivative of KT2440 with multiple *attB* sites for compatibility with SAGE, was provided by Adam Guss. The strain was routinely cultured at 30°C on lysogeny broth (LB) agar plates or in LB liquid medium. Antibiotics were added where appropriate to following final concentrations: kanamycin sulfate (50 μg/mL), apramycin sulfate (50 μg/mL), carbenicillin (50 μg/mL), gentamicin sulfate (15 μg/mL), neomycin sulfate (10 μg/mL), or spectinomycin/streptomycin (200/300 mg/L, respectively). Non-canonical amino acids 4-azido-L-phenylalanine (AzF), acetyl-L-lysine (AcK), and N6-((prop-2-yn-1-yloxy)carbonyl)-L-lysine hydrochloride (PlK) were purchased from Chem Impex, 4-Fluoro-L-phenylalanine (4FF), *p*-chlorophenylalanine (4ClF), 4-benzoyl-L-phenylalanine (BzF), *p*-acetylphenylalanine, and 4-propargyloxy-L-phenylalanine were purchased from Cayman Chemicals, and N6-((2-azidoethoxy)carbonyl-L-lysine was purchased from Sigma Aldrich. Aromatic ncAAs were dissolved in equimolar NaOH to make 100 mM stock solutions. Lysine derivative ncAAs were dissolved in ddH_2_O to make 200 mM stock solutions. Solutions of antibiotics and ncAAs were filtered through 0.22 μm sterile membrane filters.

### General culturing conditions and media

The plasmids and strains used in this study are listed in Tables S1 and S2 respectively. Routine cultivation of *E. coli* for plasmid construction and maintenance was performed at 37°C using LB (Lennox) medium supplemented with antibiotics. LB (Lennox) was used for routine pseudomonad strain maintenance and competent cell preparations. LB (Lennox) or R2A medium was used for routine *R. jostii* strain maintenance and competent cell preparations. All solid media were prepared by addition of agar (15 g/L). MOPS [3-(N-morpholino)propanesulfonic acid]-based mineral medium (MME) supplemented with 10 mM NH_4_Cl, and 10 mM glucose was used for pseudomonad GCE experiments. MME contains 9.1 mM K_2_HPO_4_, 20 mM MOPS, 4.3 mM NaCl, 20 mM NH_4_Cl, 0.41 mM MgSO_4_, 68 μM CaCl_2_, and 1× MME trace minerals (pH adjusted to 7.0 with KOH). MME trace mineral stock solution (1,000×) contains per liter 1 mL of concentrated HCl, 0.5 g of Na_4_EDTA, 2 g of FeCl_3_, and 0.05 g each of H_3_BO_3_, ZnCl_2_, CuCl_2_⋅2H_2_O, MnCl_2_⋅4H_2_O, (NH_4_)_2_MoO_4_, CoCl_2_⋅6H_2_O, and NiCl_2_⋅6H_2_O. An MME variant (MMV) additionally containing 10 mg each of niacin, pantothenate, lipoic acid, p-aminobenzoic acid, thiamine (B1), riboflavin (B2), pyridoxine (B6), and cobalamin (B12), and 4 mg each of biotin and folic acid per liter was used for *R. jostii* assays. All pseudomonad and *R. jostii* cultures were incubated at 30°C unless otherwise indicated. Cultures were incubated in 24-well deep-well plates with shaking at 700 rpm in a heated plate shaker (Southwest Science).

### Plasmid construction

Q5 High-Fidelity DNA Polymerase (New England Biolabs (NEB)) and primers synthesized by Eurofins Genomics were used in all PCRs related to plasmid construction. OneTaq DNA polymerase (NEB) was used for colony PCR. For quantitative PCR (qPCR), EvaGreen dye (Biotium) was added to reactions at a final concentration of 1×.

Plasmids were constructed using either NEBuilder HiFi DNA Assembly Master Mix (NEB) or T4 DNA ligase (NEB). Assemblies were transformed into chemically competent *E. coli* NEB 5-alpha F’IQ (NEB) using standard transformation protocols. Transformants were selected on LB (Lennox) agar containing the appropriate antibiotic and incubated at 37°C. Template DNA for PCR was synthesized by Twist Bioscience.

DNA fragments were purified from agarose gels using the Zymoclean Gel DNA recovery kit (Zymo Research). Plasmid DNA was isolated from *E. coli* using either the GeneJet Plasmid Miniprep Kit (Thermo Fisher Scientific) or the ZymoPURE II Plasmid Midiprep Kit (Zymo Research). Plasmid sequences were verified by Sanger sequencing (Eurofins Genomics) or Nanopore sequencing by (Plasmidsaurus). All plasmids used in this work are listed in Table S1, and maps of plasmids constructed in this study can be found in File SP1.

### Strain construction

All genome modifications were carried out using SAGE, in which non-replicating plasmids are integrated into the chromosome by serine recombinases. In brief, ~100 ng of non-replicating helper plasmids expressing the Bxb1, R4, or TG1 serine recombinase were co-electroporated into competent cells with cargo-containing, non-replicating *target* plasmids that contain the cognate *attP* sequence for one of the three recombinases. Upon co-electroporation, transient expression of the recombinase enables unidirectional recombination between the *attP* sequence on the target plasmid and its cognate *attB* sequence in the host chromosome. Parent strains used for this study each include an array of *attB* sequences and were constructed in prior studies (39, 41, 59). These include *Pseudomonas putida* KT2440 (AG5577), *Pseudomonas fluorescens* SBW25 (JE4621), *Pseudomonas frederiksbergensis* TBS10 (JE5041), *Pseudomonas facilor* TBS28 (RS175), and *Rhodococcus jostii* RHA1 (AG5879). Plasmids were introduced by electroporation into electrocompetent cells using previously described protocols (39). Primers used to screen for integration of the poly-*attP* cassette insertions in Pseudomonads and *R. jostii* can be found in Table S3.

### GCE efficiency assays

Starter cultures of pseudomonads and RHA1 were prepared by inoculating 6 mL of LB (pseudomonads) or R2A (RHA1) with strains from glycerol stocks. Cultures were grown at 30°C with shaking to stationary phase. These stationary-phase cultures were then diluted 1:100 into 6 mL of MME supplemented with 10 mM NH4Cl and 10 mM glucose and incubated again at 30°C with shaking until saturation. The resulting saturated cultures were used to inoculate GCE efficiency assays.

All GCE assays were performed in 24-well deep-well plates at 30°C with shaking (700 rpm, 1-mm orbital). The assay medium (MME-GCE) consisted of MME with 10 mM NH4Cl, 10 mM glucose, and a 1 × 18-amino acid solution (32). Each strain was tested in quadruplicate under two conditions: with and without ncAA supplementation (+/− ncAA). For –ncAA cultures, no ncAA was added. For +ncAA cultures, aromatic ncAAs were added to 1 mM final concentration and lysine derivatives to 10 mM.

Saturated precultures, prepared as described above, were used to inoculate 2 mL of assay medium at a 1:100 dilution. Cultures were incubated for 24 h, then diluted 1:100 in 1× PBS, and ncAA-dependent fluorescence was measured on a NovoCyte flow cytometer (Acea Biosciences). sfGFP fluorescence was recorded using a 488-nm laser and a 530/30-nm filter. Preliminary gates of 2,500 (FSC) and 10 (SSC) were applied, and 25,000 events were collected per sample.

### Creation of promoter-RBS library

A set of 449 synthetic and natural promoter sequences (Table S1) was synthesized and combined with 1 of 106 synthetic ribosome binding sites (RBSs) designed using the RBS calculator (Table S2) (77). Synthetic promoters included commonly used synthetic biology promoters and custom designs generated for this study. During cloning, each promoter was paired with a downstream synthetic RBS chosen to collectively span a ~441-fold range of predicted translation initiation rates. Promoter sequences and their flanking cloning regions were synthesized as an oligonucleotide pool by Twist Bioscience.

To construct the *Mj-TyrRS* expression library, the backbone of pJE2027 was amplified with primers oPNL2125/oPNL2126 to remove the existing tac promoter and RBS. The promoter–RBS library was amplified from the oligo pool using forward primer oPNL2127 and an equimolar mixture of six reverse primers. Each reverse primer contained: (i) a priming region specific to the oligo pool, (ii) one of six sets of degenerate RBS sequences, and (iii) a region homologous to the plasmid backbone. Both the plasmid backbone and the promoter/RBS pool were amplified using Q5 High-Fidelity DNA Polymerase (NEB). PCR products were assembled with NEBuilder HiFi DNA Assembly Master Mix (NEB) and transformed into chemically competent *E. coli* NEB 5-alpha F’IQ cells. Cells were recovered in 1 mL SOC at 37°C for 1 h. A 10-μL aliquot of the recovery culture was diluted and plated to estimate cloning efficiency and library size, while the remaining 990 μL was used to inoculate 50 mL LB with apramycin sulfate (50 μg/mL) and grown overnight at 37 °C, 200 rpm. The resulting plasmid library contained ~209,000 transformants. Plasmid DNA was then purified using a ZymoPURE II Midiprep Kit (Zymo Research) for subsequent transformation into the final host strain.

For chromosomal integration, the plasmid pool was inserted into the R4 attB site of *P. putida* strain AG5577 harboring a 2x tRNA cassette (pEVF108). Co-electroporation was performed with 500 ng of the plasmid pool and 3 μg of pGW39 (R4 integrase expression plasmid) into 50 μL of electrocompetent cells. After electroporation, cells were resuspended in 950 μL SOC and recovered at 30°C for 1 h with shaking. As above, 10 μL of the recovery culture was diluted and plated on LB agar with apramycin sulfate (50 μg/mL) to quantify the integrated library size, and the remaining 990 μL was used to inoculate 50 mL LB + apramycin (50 μg/mL), grown overnight at 30°C, 200 rpm. Library cultures were then adjusted to OD_600_ = 1, mixed with glycerol to a final concentration of 12.5%, and aliquoted (1 mL) for storage and subsequent experiments.

### Identification of active GCE-SAGE system with FACS-based selection

A FACS-based strategy was used to enrich and refine the *P. putida* promoter library based on sfGFP expression. The promoter library was introduced into *P. putida* KT2440 using SAGE, and glycerol stocks of the library were revived for selections. This was done by inoculating 50 mL of LB media with one 1-mL aliquot of the prepared glycerol stocks and incubating at 30°C for at least 6 h. Afterward, the culture was washed in MME (1×, 4 min at 5,000 rcf) and resuspended in 50 mL MME with 10 mM glucose and incubated overnight at 30°C overnight.

This overnight culture was used to inoculate a 24-well deep well plate containing 2 mL MME-GCE with 10 mM NH4Cl, 10 mM glucose, and a 1 × 18-amino acid solution as described above. This block contained wells with and without 1 mM pAzF for negative and positive selections of the library. The plate was also inoculated with strains harboring GCE v2 in the presence and absence of ncAA to provide fluorescent benchmarks for gating negative and positive events. Finally, the plate contained the parent strain AG5577 to establish background fluorescence. The plate was incubated overnight with shaking at 30°C (800 rpm, 1-mm orbital).

### Positive selections

A Sony SH800 cell sorter was used for all sorting experiments with a four-laser setup (405, 488, 561, and 638 nm) and a 100-μm sorting chip. The samples were sorted using the “Normal” sort mode into two-way 1.5-mL tubes using a forward scatter (FSC) threshold value of 0.05%. Initial gates were established on forward scatter—area by back scatter—area (FSC-A × BSC-A) and then forward scatter—height by forward scatter—width (FSC-H and FSC-W) to select for cells of expected size. The third gate was established with both the parent strains and the −ncAA condition for GCE v2.0 as a low-fluorescence control. Finally, a fourth gate was drawn using the +ncAA condition to map out the range of GCE-produced fluorescence.

With these gates, the library incubated in the presence and absence of ncAA was analyzed. A ncAA-insensitive high fluorescence peak was observed in both populations, indicative of library members that may dysregulate GCE systems and charge native amino acids. A gate capturing the top 1.5% of events for the +ncAA library but avoiding the ncAA-insensitive events was drawn, and 10,000 of those events were bulk-sorted into a 1.5-mL Eppendorf tube for a positive selection. Then, 1 mL of LB media was added to the tube, and the positive library was incubated at 30°C to recover for 2 h. After, 50 mL of MME with 10 mM glucose was inoculated with the recovered library and cultured for 48 h at 30°C.

### Negative selections

After incubation, the positive library was sub-cultured into fresh MME with 10 mM glucose at a 50-fold dilution and incubated overnight with shaking at 30°C. This culture was analyzed by the Sony SH800 cell sorter using gates drawn for low background events from the parent strain and GCE v2.0 in −ncAA condition. Low background events were single cell sorted with high purity into a 96-well plate containing 150 μL of LB media. Controls, including the parent strain, and GCE v2.0 were also added to the plate to provide direct comparisons during validation. The plate was incubated with shaking at 30°C overnight and then used to inoculate a 96-deep well plate for glycerol stocks as well as a 96-well plate containing 150 μL of MME with 10 mM glucose for validation assays.

### Validation experiments

After incubation overnight at 30°C with shaking, the above plate was used to inoculate two additional black-walled, clear-bottom 96-well microplates, one containing 150 μL of MME-GCE without ncAA to determine background incorporation of the potential hits and one with MME-GCE containing 1 mM pAzF to determine GCE efficiency. These plates were incubated for 24 h using the “fast shaking” 1 mM double orbital setting on a Neo2SM plate reader (BioTek).  Fluorescence (*F*_501,520_ for sfGFP) was then measured for both plates. Background fluorescence readings from wells containing the non-fluorescent parent strain were averaged and subtracted from sample readings before analysis. A subset of potential hits from the initial block were then screened in a typical GCE experiment using 2 mL of culture (see above), and promising clones were sequenced.

### Expression and purification of ncAA-protein for mass spectrometry analysis

To produce sufficient protein for mass spec validation, 5 mL LB cultures were inoculated from frozen glycerol stocks and cultured overnight at 30°C. Overnight cultures were subcultured into 5 mLs MME with 10 mM NH4Cl and 10 mM glucose and cultured overnight at 30°C. These cultures were used to inoculate 200 mL of MME-GCE with ncAA in a 1-L Erlenmeyer flask at a 1:100 dilution. These cultures were grown overnight at 30°C with shaking. The next day, the cultures were pelleted by centrifugation (15 min, 5,500 rcf), and spent media were removed. The cell pellets were resuspended in a lysis/wash buffer (50 mM Tris-HCl, 500 mM NaCl, and 5 mM imidazole [pH 7.5]) and lysed via sonication. Cell debris was pelleted at 15,000 rcf for 25 min at 4°C and clarified cell lysate was recovered. To bind His_6_-tagged protein, clarified cell lysate was incubated with 200 μL of Ni-NTA resin (HisPur Ni-NTA Resin, Thermo Scientific) at 4°C for 1 h with rocking. Resin was collected and extensively washed with 50 resin bed volumes (bv) of lysis buffer. Bound protein was eluted from the resin by incubation with five bed volumes of elution buffer (lysis buffer with 200 mM imidazole), concentrated, and exchanged into storage buffer (50 mM phosphate, 150 mM NaCl) with a 3-kDa MWCO Vivaspin spin-concentration filter (GE Health Sciences). Protein concentration was determined by absorbance at 280 nm, flash-frozen with liquid nitrogen, and stored at −80°C until needed. For purification of sfGFP,_2XpAzF_ an additional size exclusion chromatography step was added (Superdex 200 increase 10_300 column from Cytiva, Product #28990944, PBS Buffer pH 7.4, Flow rate 0.5 mL/min). However, during analysis, sfGFP_2XpAzF_ experienced some proteolytic degradation, though maintained its fluorescent signature (Fig. S2).

For mass analysis of protein, the purified proteins were then analyzed by Creative Proteomics facility on a Synapt G2-Si mass spectrometer by the following method. The protein of interest was isolated using chromatographic techniques (C4 on the H-Class UPLC) and introduced to the Synapt G2-Si using a dual orthogonal API source (Lock-Spray ESI/APCI) and subjected to CID fragmentation. The mass spectrometer was operated in ESI positive resolution mode in the range 300–5,000 Da. The capillary voltage was set to 3.0 kV with a sampling cone voltage of 78 V, a source temperature of 150°C, and a desolvation temperature of 550°C. The cone gas was set to 0 L/h with desolvation gas at 300 L/h, and the nebulizer set to 6.0 bar. The protein’s molecular weight can then be determined by deconvoluting the fragment masses using Waters MaxEnt software.

### Statistical analysis

All reported values represent the mean of four independent experimental replicates. Error bars correspond to the standard deviation (SD) calculated from these replicates. No additional statistical tests were applied unless otherwise indicated.

## Data Availability

All data are available in the main text or the supplemental materials.

## SUPPLEMENTAL MATERIAL

File SP1 mbio.01273-26-s0001.csv DNA sequences corresponding to plasmids listed in Table S4.

Supplemental Material mbio.01273-26-s0002.docx Fig. S1 to S7; Tables S4 to S5.

Table S1 mbio.01273-26-s0003.xlsx Promoter sequences found in the combinatorial library employed in library screens.

Table S2 mbio.01273-26-s0004.xlsx Ribosomal binding site sequences paired with promoters employed in library screens.

Table S3 mbio.01273-26-s0005.xlsx Highest performing promoter and RBS sequences identified by library screen.

ASM does not own the copyrights to Supplemental Material that may be linked to, or accessed through, an article. The authors have granted ASM a non-exclusive, world-wide license to publish the Supplemental Material files. Please contact the corresponding author directly for reuse.
